# Supplementary material for: Novel Genetic Variants and Clinical Profiles in Peters Anomaly Spectrum Disorders
Source: Int J Mol Sci. 2025 Jul 4;26(13):6454. doi: 10.3390/ijms26136454 (PMC12250460; doi:10.3390/ijms26136454)
Supplement: Supplementary file 1 [file ijms-26-06454-s001.zip › ijms-3625507-supplementary.pdf]

Table S1. Congenital Glaucoma and ASD associated gene list used for filtering of WES data.

ABCA1	*CRYGA*	*GSTM3*	*NT5C1B*	*SHH*
ABCB6	*CRYGB*	*GSTP1*	*NTF4*	*SHISA2*
ABCD3	*CRYGC*	*GSTT1*	*NTN1*	*SIL1*
ABHD12	*CRYGD*	*GTF2IRD1*	*OAS3*	*SIPA1L3*
ABO	*CRYGS*	*HCCS*	*OCRL*	*SIX1*
ACVR1C	*CRYZ*	*HDAC9*	*OGG1*	*SIX2*
ACYP1	*CSNK2A2*	*HEY1*	*OLFM2*	*SIX3*
ADAM9	*CTDP1*	*HEY2*	*OLFM3*	*SIX5*
ADAMTS10	*CTNNB1*	*HIP1*	*OPA1*	*SIX6*
ADAMTS16	*CTNND2*	*HRAS*	*OPA3*	*SLC16A12*
ADAMTS17	*CTTN*	*HSF4*	*OPTC*	*SLC18A2*
ADAMTS18	*CTTNBP2*	*HSPG2*	*OPTN*	*SLC1A3*
ADAMTSL1	*CXCR4*	*IARS2*	*P3H2*	*SLC33A1*
ADAMTSL3	*CYP1B1*	*IDO1*	*PAK5*	*SLC40A1*
ADAMTSL4	*CYP27A1*	*IFNGR1*	*PALLD*	*SLC4A11*
ADAMTSL4 (AR)	*CYP51A1*	*IGFBP7*	*PANK4*	*SLC4A3*
ADIPOQ	*DACH1*	*IL4*	*PARK7*	*SLC4A4*
AFAP1	*DCN*	*IMMT*	*PAWR*	*SLC6A3*
AGK	*DGKQ*	*IMPG1*	*PAX1*	*SLC7A8*
ALDH18A1	*DHCR7*	*INPP5K*	*PAX2*	*SLURP1*
ALX4	*DIP2A*	*INTS1*	*PAX3*	*SMAD2*
ANGPT1	*DLL4*	*IPO13*	*PAX6*	*SMAD4*
ANGPT2	*DMPK*	*IRF4*	*PCP4*	*SMO*
ANGPTL7	*DMXL1*	*JAG1*	*PDE6B*	*SMOC2*
ANK2	*DNAJB1*	*JAM3*	*PDLIM3*	*SNCG*
ANO7	*DNASE2B*	*JUN*	*PEX1*	*SOD2*
ANPEP	*DNM2*	*KCNA4*	*PEX10*	*SORBS2*
AP4B1	*DNMBP*	*KCNJ13*	*PEX11B*	*SOX1*
APOD	*DOCK5*	*KERA*	*PEX13*	*SOX2*

APP	*DPT*	*KIAA1109*	*PEX14*	*SOX3*
ARHGEF10L	*DST*	*KLHL2*	*PEX2*	*SPARC*
ARID1B	*DSTN*	*KRT12*	*PEX3*	*SPARCL1*
ARPC1A	*DYNC1H1*	*LAMB1*	*PEX6*	*SPATA13*
ARPC2	*EEF1A1*	*LAMB2*	*PGAM1*	*SPP1*
ARPC3	*EFEMP1*	*LAPTM4B*	*PGAM5*	*SQSTM1*
ASB10	*EFNA5*	*LCA5*	*PHIP*	*SRD5A3*
ASPH	*EID1*	*LCT*	*PIGY*	*SRSF5*
ATAD3A	*EIF1AX*	*LDB1*	*PIK3R1*	*STX3*
ATOH7	*EIF2B2*	*LEF1*	*PINK1*	*SUCLG1*
ATP2B1	*EIF4G3*	*LEMD2*	*PITX2*	*SVEP1*
ATP2B2	*ELN*	*LGSN*	*PITX3*	*SYF2*
ATP2B3	*EPHA2*	*LIM2*	*PKN1*	*SYNM*
ATP2B4	*EPHX1*	*LMX1B*	*PLOD1*	*TAF1A*
ATXN2	*ERCC2*	*LONP1*	*PNPT1*	*TAL1*
B3GLCT	*ERCC8*	*LOXL1*	*POLG*	*TAPT1*
B4GALT3	*ERRFI1*	*LRP2*	*POMGNT1*	*TBK1*
B4GALT7	*ESCO2*	*LRP6*	*POMT1*	*TCF7L1*
B4GAT1	*ESYT3*	*LRRC4*	*POU5F1*	*TCF7L2*
BACH2	*EXO5*	*LSS*	*PPP3R1*	*TDRD7*
BCO2	*EXOSC10*	*LTB*	*PRDM5*	*TECTA*
BCOR	*EYA1*	*LTBP2*	*PRODH2*	*TEK*
BDNF	*EZR*	*LUM*	*PROX1*	*TFAP2A*
BEST1	*FAM120A*	*MAB21L2*	*PRPF8*	*TGFBI*
BFSP1	*FAM126A*	*MACF1*	*PRSS56*	*TH*
BFSP2	*FAM27E5*	*MAF*	*PRX*	*THBS1*
BIN3	*FAR1*	*MAFA*	*PTCH1*	*THBS2*
BMP4	*FBN1*	*MAP6*	*PTGR2*	*TIMP1*
BMP7	*FGF2*	*MED13*	*PTMA*	*TIMP2*
BRD4	*FGFR2*	*MEF2B*	*PTPN11*	*TLX1*

BTAF1	*FLNA*	*MFAP2*	*PXDN*	*TMCO1*
CALD1	*FLNB*	*MFN2*	*RAB3GAP1*	*TMCO3*
CAPZA1	*FN1*	*MFRP*	*RAD54B*	*TMED10*
CARD10	*FNDC3B*	*MGP*	*RAMP2*	*TMEM51*
CC2D2A	*FOS*	*MIP*	*RBM25*	*TMEM70*
CCN2	*FOXA2*	*MIR182*	*RECQL4*	*TMSB4X*
CCND2	*FOXC1*	*MITF*	*RELN*	*TMTC1*
CD5	*FOXC2*	*MLH3*	*RERE*	*TOR1AIP1*
CDKN2B	*FOXE3*	*MMP1*	*RGS1*	*TP53*
CFL2	*FOXF2*	*MMP12*	*RGS2*	*TP53BP2*
CHD7	*FOXH1*	*MMP13*	*RGS6*	*TPM1*
CHI3L1	*FTL*	*MMP14*	*RGS7*	*TPT1*
CHMP4B	*FYCO1*	*MMP2*	*RGS9*	*TRIM11*
CHRDL1	*FZD5*	*MMP3*	*RIC1*	*TRIM44*
CLPB	*GAL3ST3*	*MMP9*	*RNF149*	*TRNT1*
CNBP	*GALC*	*MSX2*	*ROR2*	*TRPM3*
CNGB3	*GALE*	*MTA1*	*RPE65*	*TUBA1A*
CNN3	*GALK1*	*MVK*	*RPGRIP1*	*TUBB*
CNR2	*GALT*	*MYB*	*RPL11*	*TULP2*
CNTN4	*GAS7*	*MYH9*	*RPL12*	*TULP3*
COL11A1	*GATA6*	*MYL6*	*RPL23*	*TXNRD2*
COL18A1	*GBA2*	*MYOC*	*RPL23A*	*TYR*
COL1A1	*GCG*	*MYOG*	*RPL27*	*UCHL1*
COL1A2	*GCM2*	*NACA*	*RPL31*	*UGT1A1*
COL2A1	*GCNT2*	*NACC1*	*RPL37*	*UGT1A6*
COL3A1	*GDF3*	*NCOA6*	*RPL37A*	*UGT1A9*
COL4A1	*GDF6*	*NDP*	*RPL41*	*UGT2B7*
COL4A2	*GDNF*	*NDUFA5*	*RPL5*	*UNC45B*
COL4A3	*GEMIN4*	*NECAP2*	*RPL6*	*UQCRB*
COL4A4	*GFER*	*NECTIN3*	*RPL7*	*VCAN*

COL4A5	*GIPC1*	*NEU1*	*RPL9*	*VDAC2*
COL7A1	*GJA1*	*NEUROG2*	*RPS20*	*VIM*
COL8A2	*GJA3*	*NF1*	*RPS24*	*VLDLR*
COMMD1	*GJA8*	*NF2*	*RPS25*	*VSX2*
COMT	*GLA*	*NFATC1*	*RPS27A*	*WDR36*
COX7C	*GLI2*	*NFE2*	*RPS3A*	*WDR87*
CPAMD8	*GNAS*	*NHS*	*RPS8*	*WFS1*
CREBBP	*GNB5*	*NOS3*	*RRAGA*	*WNT7A*
CRIM1	*GNPAT*	*NOTCH2*	*RRM2B*	*WRN*
CRPPA	*GPATCH3*	*NPC2*	*RYR1*	*XKR4*
CRYAA	*GPM6A*	*NPHP1*	*SALL2*	*XRCC1*
CRYAB	*GPR12*	*NPHS1*	*SBF2*	*XYLT2*
CRYBA1	*GPR161*	*NPHS2*	*SC5D*	*ZNF185*
CRYBA2	*GPR180*	*NPRL3*	*SCHIP1*	*ZNF350*
CRYBA4	*GPX1*	*NPS*	*SEMA3E*	*ZNF410*
CRYBB1	*GSN*	*NR4A2*	*SERPINF1*	*ZNRF1*
CRYBB2	*GSTA1*	*NRCAM*	*SGCE*	
CRYBB3	*GSTM1*	*NSD2*	*SH3PXD2B*	
